# Accuracy of Wireless Hand-Held Guided Ultrasound Injections in the Trapeziometacarpal Joint: A Cadaveric Study

**DOI:** 10.7759/cureus.45779

**Published:** 2023-09-22

**Authors:** Ceyran Hamoudi, Antoine Martins, Thibault Willaume, Pierre-Antoine Debordes, Philippe Liverneaux, Sybille Facca

**Affiliations:** 1 Department of Hand Surgery, University Hospital of Strasbourg, Strasbourg, FRA; 2 Department of Hand Surgery, Private Hospital La Châtaigneraie, ELSAN, Beaumont, FRA; 3 Department of Radiology, University Hospital of Strasbourg, Strasbourg, FRA; 4 Orthopedics, Strasbourg University, Strasbourg, FRA

**Keywords:** ultrasound-guided imaging, blinded, trapeziometacarpal joint, injection, hand-held ultrasound

## Abstract

Background

Symptomatic trapeziometacarpal osteoarthrosis can be treated with an ultrasound-guided injection in the early stages. This cadaveric study aimed to assess the hypothesis suggesting enhanced accuracy and reliability of hand-held ultrasound (HHUS) injections compared to blind injections into the trapeziometacarpal joint (TMC).

Materials and method

Our series included 20 fresh cadaveric hands, with a total of 20 TMC randomly assigned to two groups. In group A, 10 TMC received a blinded injection, and in group B, 10 TMC received an ultrasound-guided injection with HHUS. Methylene blue was injected, and anatomical dissection was performed to assess the intra-articular location of the dye. The injection was considered accurate if the intra-articular synovial fluid was stained after opening the articular capsule on the dorsal approach. If there was no injection, it was inaccurate. A statistical analysis was performed, and p <.05 indicated a significant difference.

Results

Two thumbs were excluded during the study due to an existing trapeziectomy. In group A, 10 blind injections of TMC were performed, with 70% (7/10) of injections graded as accurate. In group B, eight ultrasound-guided injections were performed, with 75% (6/8) achieving accuracy. A Fisher's exact test was performed, and the results indicated no statistically significant difference in injection accuracy between the two groups (P = 1, odds ratio = 0.788).

Conclusion

Hand-held ultrasound guided TMC injections were not more accurate than blind injections performed by an experienced hand surgeon. Nonetheless, additional studies with a larger sample and comparative studies with conventional cart-based machines are necessary to evaluate the potential of this newly accessible device.

## Introduction

Symptomatic trapeziometacarpal osteoarthrosis (TMO), also known as rhizarthrosis, is a frequent pathology associated with significant hand disability. It has a reported incidence of 3% in men and 5% in women among Caucasians [1,2]. This condition is more common among postmenopausal women; nevertheless, even with radiological findings of TMO, most of them remain asymptomatic [3]. When the trapeziometacarpal joint (TMC) becomes symptomatic, patients often experience pain at the base of their thumb when performing pinching and gripping activities [4]. The importance of addressing symptomatic TMO lies in the impact it has on an individual's quality of life and functionality. In some cases, pain may extend to the thenar region or the metacarpophalangeal joint. As the condition progresses, the thumb may assume a hyperextended position at the metacarpophalangeal joint, associated with an adducted first metacarpal bone, further compromising hand function and causing chronic discomfort.

Conservative treatments for TMO include activity modification, analgesics, nonsteroidal anti-inflammatory drugs (NSAIDs), splinting, hand therapy, and intra-articular injections [5]. Some authors recommend intra-articular injections of corticosteroids or hyaluronic acid [6], with studies suggesting greater effectiveness when performed under ultrasound guidance in cadaveric models [7].

While ultrasound-guided injections are usually performed using conventional cart-based ultrasound (US) machines, recent years have seen the development of hand-held ultrasounds (HHUS). These devices are cheaper and more readily available for clinicians compared to high-end ultrasounds. Their size, ease of use, and portability are other marketing advantages. HHUS has found a place in the diagnosis in critical care facilities where non-specialist radiologists can access it [8,9]. They have also proven valuable and cost-effective for screening purposes [10]. Nevertheless, their functionality is somewhat limited compared to the conventional US due to a limited choice of probes. Although the use of HHUS has been assessed for other medical applications [11], its role in procedural guidance remains less explored. 

In this cadaveric study, we aimed to compare the added benefit of using wireless HHUS guidance versus that of blind injection in the TMC joint in terms of accuracy. Our hypothesis was that injections under HHUS guidance would be more accurate than blind injections. The null hypothesis posits that there is no significant difference between the two groups.

## Materials and methods

Twenty fresh upper extremities from adult cadavers of Caucasian origin were obtained from the voluntary body donation program of the IRCAD Institute (Institut de recherche contre les cancers de l'appareil digestif) according to the legal procedures and ethical framework governing body donation programs in France. All specimens were fresh and frozen. Previous medical history was unknown [12].

A total of 20 hands were included due to constraints of cadaver availability and research resources, labeled from 1 to 20, and then randomly assigned to two groups using a number randomizer website (www.randomizer.org) to ensure unbiased group selection. Ten hands were assigned to group A (the blind injection technique) and ten hands to group B (the HHUS-guided injection technique). Approximately 2 mL of methylene blue dye was injected through a 22-gauge in-needle in both groups [12].

**Figure 1:**
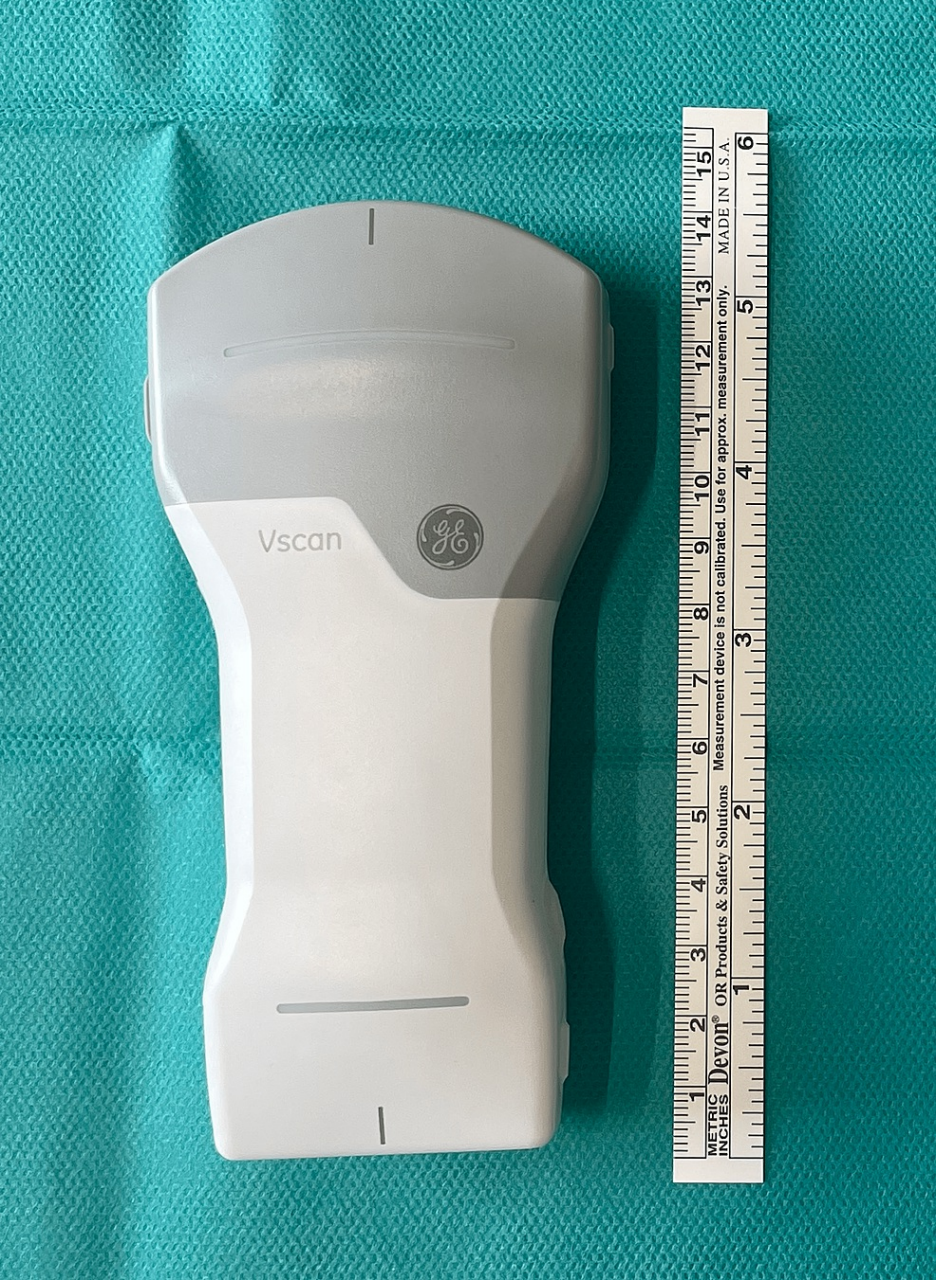
Vscan air CL ultrasound (GE Healthcare, Norway)

In group A, blind injections of TMC were performed by an experienced senior level 3 hand surgeon (A.M.) [13]. In group B, ultrasound-guided injections were executed by different experienced senior level 5 hand surgeons with a background in musculoskeletal sonography (S.F.) [13]. A Vscan Air CL ultrasound (GE Healthcare, Norway) equipped with one linear probe (3 to 12 MHz) was used paired with a tablet computer (iPad, Apple, California, US) and an interface gel (Uni’gel US1, Asept InmedTM, Quint Fonsegrive, France) [12] (Figure 1).

Ethical approval by the institutional review board was not required for this cadaveric study [12].

Injection technique in TMC

**Figure 2:**
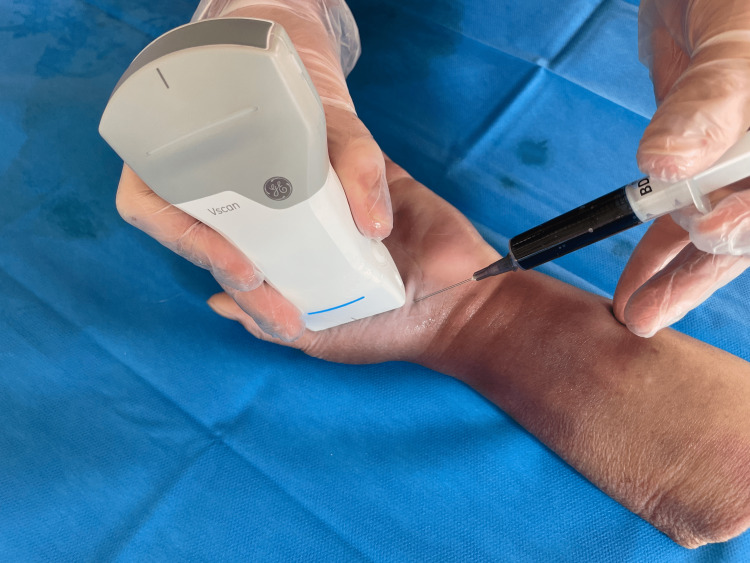
Injection technique of the TMC

In group A, the injection was administered by the volar approach, and the interval between the base of the first metacarpal bone and the trapezium was searched by the palpation method. The needle was inserted in line with the axis of the second metacarpal bone, and the dye was administered (Figure 2).

**Figure 3:**
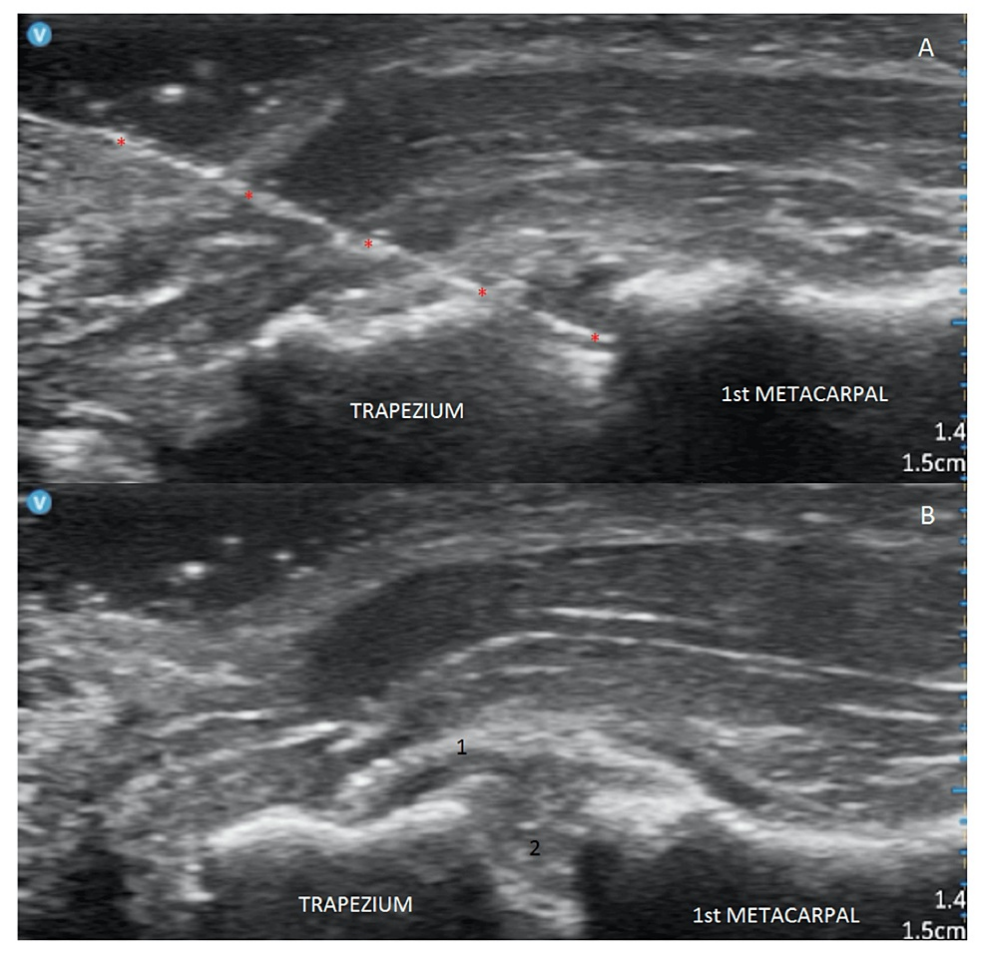
Sagittal views of the TMC (A) HHUS-guided injection of the TMC. Red stars show the needle. (B) Sagittal view of the TMC after dye injection. Number 1 = joint capsule. Number 2 = TMC joint with dye effusion.

In group B, the sagittal view was used to identify the TMC joint, and the injection was performed on an in-plane long axis centered on the axis of the first metacarpal bone over the proximal part of the thenar eminence. Real-time visualization and precise control of the injection site and depth were maintained to ensure accuracy. Detailed records of the HHUS-guided injections were recorded (Figure 3).

Anatomic dissection

Dissection was performed after injection in all anatomical pieces by a different examiner (C.H.) to assess the intra-articular location of the dye. Another examiner, independent of our department and not involved in our study, assessed each dissection and its results [12]. A surgical approach to the TMC was performed by a dorsal approach; subcutaneous tissues and intra-articular content were assessed.

Evaluation of the accuracy

**Figure 4:**
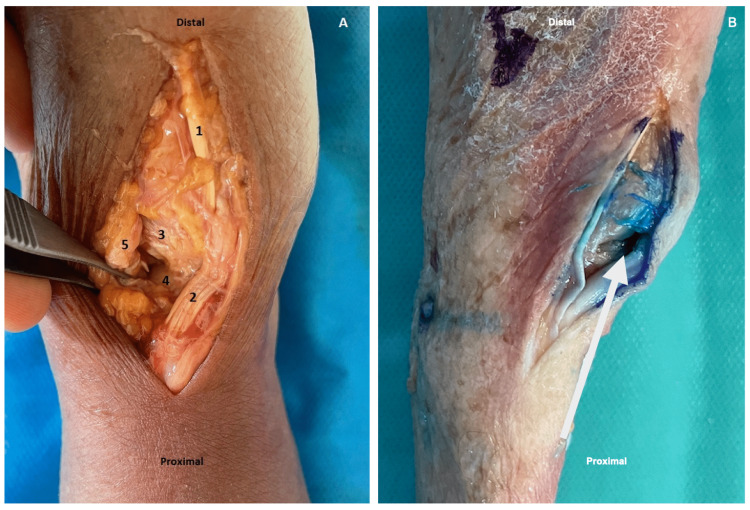
Accuracy of injection according to dye diffusion after anatomical dissection. (A) Injection not accurate = dye is not present inside the TMC joint. 1 = abductor pollicis longus, 2 = extensor pollicis brevis, 3 = base of the 1st metacarpal bone, 4 = trapezium, 5 = joint capsule. (B) Accurate injection = dye is present inside the TMC joint. The white arrow indicated intra-articular dye.

Injection was considered accurate if intra-articular synovial fluid was stained after opening the articular capsule on the dorsal approach. Injection was not accurate if none (Figure 4).

Statistical analysis

The statistical analysis of the data collected in this study included categorical variables described using counts and proportions. The comparison of injection accuracy (accurate vs. non-accurate) for the trapeziometacarpal joints was performed using a Fisher exact test. A p-value of <0.05 was considered statistically significant.

All statistical analyses were performed using R software version 4.1.1. R Core Team (2021). R: A language and environment for statistical computing. R Foundation for Statistical Computing, Vienna, Austria (URL https://www.R-project.org/).

## Results

In total, 18 hands were used for this study, two of which were excluded because of an existing trapeziectomy. The results are summarized in Table 1.

**Table 1:** Comparison of injection accuracy between blind injections (group A) and ultrasound-guided injections (group B) based on the presence of intra-articular dye in the TMC joint

Group A			Group B		
Cadaveric hands (n)	Side (L/R)	Accuracy of injection	Cadaveric hands (n)	Side (L/R)	Accuracy of injection
1	L	Accurate	1	L	Accurate
2	L	Accurate	2	R	Accurate
3	L	Not accurate	3	L	Accurate
4	L	Accurate	4	R	Not accurate
5	L	Accurate	5	L	Excluded
6	R	Accurate	6	L	Accurate
7	L	Accurate	7	R	Accurate
8	R	Not accurate	8	R	Not accurate
9	L	Not accurate	9	L	Excluded
10	R	Accurate	10	R	Accurate

In total, 10 TMC were included in group A, and eight TMC were included in group B.

In group A, 70% (7/10) of injections were graded as accurate (95% CI: 44.0% to 89.0%) and 30% (3/10) as not accurate (95% CI: 11.0% to 56.0%). In group B, 75% (6/8) were classified as accurate (95% CI: 44.5% to 94.5%), and 25% (2/8) were classified as not accurate (95% CI: 5.5% to 55.5%).

A Fisher's exact test was performed, and the results indicated no statistically significant difference in injection accuracy between the two groups (p = 1, odds ratio = 0.788). Therefore, we do not have sufficient evidence to reject the null hypothesis, suggesting that the method of injection does not significantly influence the accuracy of injection in this context.

## Discussion

The trapeziometacarpal joint (TMC) is the second most affected joint by osteoarthritis (OA) in the hand [14]. The Framingham Osteoarthritis Study found that 6.8% of adults exhibited radiographic hand findings [1]. The etiology of trapeziometacarpal osteoarthritis (TMO) is complex. Some authors have speculated that the prevalence in women can be explained by a smaller articular surface, resulting in higher stress forces on the TMC [15]. Joint laxity has also been incriminated; it leads to TMC subluxation and, thus, the incongruence between the trapezium and the base of the first metacarpal bone [16]. The aging population and the increasing prevalence of obesity are also implicated in the development of osteoarthrosis [17]. Regardless of the etiopathogenesis, a range of conservative treatments are available for clinicians as the first-line treatment for TMO, including activity modification, analgesics, anti-inflammatory drugs, physiotherapy, and hand splinting. When those fail, corticosteroids or hyaluronic acid intra-articular can be used to treat pain and disability in the hands [6]. Intra-articular injection can be performed with fluoroscopic X-ray guidance to control the location of the needle before injection of the TMC. Helm et al. reported in an uncontrolled prospective study of 60 patients that only in 58% of cases was the joint space correctly injected [18].

Ultrasound guidance has emerged as the gold standard for administering steroid injections; nevertheless, evidence supporting its use remains limited [19]. Many available studies are either uncontrolled prospective trials or are conducted on cadaveric specimens [19]. The consensus is that US guidance offers advantages over blind or fluoroscopy-guided injections. Its use enhances accuracy by enabling direct visualization of the targeted anatomical structure, thereby reducing the associated risk of nerve or vascular punctures and tendon damage [20]. Additionally, it is less harmful to the patient, as there is no exposure to ionizing radiation and no need for an iodine-based contrast medium [20]. Umphrey et al. reported a 94% intra-articular accuracy rate using ultrasound-guided injections in a cadaveric study; 16 of 17 injections entered the TMC [7].

Conventional cart-based ultrasound devices usually carry out the guidance; they offer a wide range of modalities and generate high-quality images. However, the price of those high-end ultrasounds and their lack of portability can be a limitation for some facilities and clinicians. Recently, ultrasound devices have become miniaturized, cheaper, and more readily available in clinical practice. Hand-held ultrasounds (HHUS) have been developed in that way; their size is significantly lower, they can be carried in the physician’s lab coat, and they have a significantly lower price, creating opportunity.

Numerous devices are available for clinicians seeking to integrate POCUS into their clinical routines. Each device comes with different options; some are linked to smartphones or screen units, while others operate wirelessly, as used in our study. However, we are unaware whether their added benefits in procedural guidance have been assessed in other studies regarding the TMC-guided-injection.

HHUS effectiveness in evaluating the musculoskeletal system has been explored by Falkowski AL et al. in 100 patients, comparing HHUS to conventional cart-based ultrasound [21]. The most common joint evaluated was the shoulder, and they found that the results from HHUS were concordant or discordant without clinical relevance in 96% of cases (96/100). Moreover, Falkowski AL et al. described a low color doppler sensitivity of the HHUS compared to cart-based ultrasound. However, the additional finding of hyperemia did not remarkably change clinical management [21].

In the case of TMO, classical US findings are joint effusion, a diminution of the joint space, an erosive aspect of the articulation, and osteophytes [22]. Whenever osteophytes are present in advanced TMC arthritis, they make locating the joint challenging and may contribute to blind injection failure. In our experience, the osteophytes in TMO narrow the joint space on its dorsal aspect and open it on its volar side. Moreover, the radiographic stage does not necessarily correlate with the symptoms of patients; therefore, with various TMO clinical presentations, US guidance may represent a viable option to avoid osteophytes while performing the injection. In our study, most cadaveric pieces did not exhibit advanced rhizarthrosis.

The initial hypothesis of our study, suggesting that injections guided by HHUS would be more accurate compared to blind injections in the TMC, was not confirmed. These results emphasize that both blind injections and hand-held ultrasound (HHUS)-guided injections can be effective treatment options for patients with TMC joint OA. This allows clinicians to select the most appropriate approach based on individual patient factors and resource availability. However, while expertise did not significantly influence injection accuracy in our study, several factors inherent to cadaveric specimens and the relatively limited scope of our investigation could have influenced the observed accuracy rates. Primarily, specimens may provide a consistent and stable anatomical environment, making it easier to target the trapeziometacarpal joint accurately, even with blind injections. Second, the absence of patient-related variables, such as pain or discomfort, which can affect patient cooperation and, consequently, injection accuracy, contributed to the consistent results.

In this study, we focused exclusively on assessing the accuracy of the TMC injection. However, the potential applications of procedural guidance using HHUS have not been fully explored in conditions such as De Quervain tenosynovitis, metacarpophalangeal joints, and proximal interphalangeal joints. However, a previous cadaveric study conducted by our team has explored the accuracy of wireless HHUS injections versus blind injections in the flexor tendon sheath (FTS). The evaluation was based on the dye’s filling pattern in the FTS, categorized as stage I (no filling), stage II (<50% filling), and stage III (>50% filling). This study did not establish the superiority of ultrasound guidance in accuracy (P = .35). Thirty-nine FTS blind injections were performed, with 82% (32/39) fingers achieving stage III filling. Forty FTS ultrasound-guided injections were performed, with 90% (36/40) of fingers achieving stage III filing [12]. Furthermore, our team conducted an additional cadaveric study to examine the feasibility and reliability of HHUS in carpal tunnel injections. Using the radial approach beneath the flexor carpi radialis with an in-plane short-axis view. We performed a total of 16 injections safely. Subsequent anatomical dissection revealed only two cases of nerve puncture without intraneural injection [23].

This study had some severe limitations. Since it is a cadaveric study, the clinical outcome cannot be measured. Moreover, the body donation program’s anonymity policy did not allow information regarding the age and gender of the specimens. Finally, the amount of injected TMC in each group is low, and therefore, definitive conclusions cannot be drawn.

To overcome the limitations of this study and advance our comprehension of TMC joint injections, several avenues for further research and improvements in study design should be explored. First, conducting additional clinical studies with a larger sample is essential. Second, explore patient-related factors, such as the presence of osteophytes and disease severity, and their impact on injection accuracy. Long-term clinical outcome assessments of patients undergoing TMC joint injections using both techniques are necessary. Finally, conducting cost-effectiveness analyses comparing blind injections and HHUS-guided injections to guide healthcare decision making.

## Conclusions

In summary, our study aimed to assess the utility of HHUS for TMC injections and its potential benefits for improving injection accuracy. Contrary to our initial hypothesis, our results did not confirm the anticipated superiority of HHUS-guided injections over blind injections in the TMC, consistent with similar findings previously reported in the context of the flexor tendon sheath. However, it is essential to emphasize that ultrasound-guided injections remain the gold standard, particularly for inexperienced operators. Moreover, HHUS has surged across numerous medical and surgical applications in recent years. It is an interesting tool for hand surgeons that has the potential to enhance diagnostic precision for specific conditions when integrated into clinical practice. Lastly, using HHUS by hand surgery fellows to navigate injections during the learning curve holds promise.
